# Supplementary material for: Are carbon emissions trading and green financial instruments synergistic? -Comprehensive quantitative research based on content analysis
Source: PLoS One. 2024 Mar 7;19(3):e0298601. doi: 10.1371/journal.pone.0298601 (PMC10919601; doi:10.1371/journal.pone.0298601)
Supplement: S1 Data — (ZIP) [file pone.0298601.s001.zip › Data/地级市Z_F工作报告.zip!/╦─┤¿/─┌╜¡╩╨/└┤╫╘╣1⁄2╓┌║┼í╛┬φ┐╦╩2╛▌═°í┐_2018.docx]

各位代表：

　　我代表市人民政府，向大会报告工作，请予审议，并请市政协委员提出意见。

一、2017年工作回顾

　　2017年，在省委、省政府和市委的坚强领导下，在市人大、市政协的监督支持下，我们紧紧围绕市七届人大二次会议确定的目标任务，坚持稳中求进工作总基调，用心、认真、尽责，敢挺、苦干、实拼，经济社会保持平稳健康发展。实现地区生产总值1332.1亿元，增长7.1%;完成地方一般公共预算收入56.1亿元，同口径增长9.1%;农村居民人均可支配收入达13640元，增长9.2%;城镇居民人均可支配收入达30393元，增长8.6%。

　　(一)坚持转方式、调结构，发展质量明显提升

　　工业经济稳中向好。规模工业增加值增长6.1%;工业入库税金增长20.4%;工业用电量增长19.7%，居全省第3位。产业结构调整步伐加快，五大特色优势产业实现产值1131亿元，高新技术和战略性新兴产业产值占规模工业总产值的比重分别达31.2%、20.6%。深入实施先进制造强市“三百行动”，发展先进制造企业30户，技改传统制造企业30户，培育制造业名优品牌31个。新增规模以上工业企业34户。服务企业力度加大，设立10亿元新兴产业股权投资基金，投入工业帮扶资金3亿多元，一批重点企业产能达到历史最好水平，34户停产半停产企业恢复生产。落实富余电、直购电、阶梯气价等政策，降低企业用电用气成本2.2亿元。内江经开区等重点园区新入驻单项投资5000万元以上的企业(项目)37个，产业集聚度达74%。科技对工业发展的带动能力增强，建成创新创业孵化载体22家，孵化面积13.5万平方米，隆昌高新技术创业服务中心成功创建国家级科技企业孵化器;6户企业被新认定为国家高新技术企业。

　　服务业加速提档升级。实现社会消费品零售总额511.9亿元，增长11.1%。实现服务业增加值461.8亿元，增长9.0%，占GDP比重提高7.6个百分点。服务业税收收入占比达到65.5%，提高2.1个百分点。新增限额以上商贸企业和规模以上服务业企业74户。8大电商集聚区入驻企业237户，全市电子商务网络交易额达到497.2亿元，保持全省第3位。编制现代物流业发展规划和行动计划，苏宁川南管理总部暨配送中心一期建成投用，全市第三方物流企业营业额增长18.1 %。深入实施旅游发展“一三八”计划，实现旅游总收入263.9亿元，增长22.1%，黄鹤湖旅游区成功创建国家AAAA级景区。大力发展金融业，实现金融业增加值48.8亿元，增长7.4%，引进域外金融机构6家，全市社会融资规模达1400亿元。

　　现代农业加快发展。“12345”现代农业产业提升行动和“351”特色农业产业发展方案有序推进，全市粮食产量达158.2万吨。“甜城味·大千故里优质农产品” 市域公用品牌入选“四川省十大优秀农产品区域公用品牌”，100万头“内江黑猪”产业化项目加快实施，优质粮油等4大功能区初具规模，5个万亩以上产业示范片新发展3.5万亩，建成全国最大的无花果连片基地、血橙基地。新增农民合作社466个、家庭农场172户，创建无公害农产品、绿色食品、有机食品、农产品地理标志249个。严格保护耕地和基本农田，全面完成永久基本农田划定任务，新建高标准农田20万亩。完成病险水库除险加固11座。

　　(二)坚持打基础、利长远，城乡面貌不断改善

　　“项目年”活动深入开展。成立项目推进中心、重点项目决策论证委员会，28个省级重点项目、287个市级重点项目完成年度投资计划，全社会固定资产投资完成860.2亿元。启动“一带一轴一区”重要交通枢纽三年推进行动，完成交通基础设施投资32.5亿元。联合水库、长桥水库、河库联网输水工程等重点水利项目加快建设，完成水利基础设施投资12.9亿元。

　　“双百”城市加快建设。坚持规划引领，编制完成专项规划18个、城市设计4个。开工建设“双百”城市项目86个，完成投资41亿元。推进 “一中心四组团”联动发展，花园滩大桥建成通车，黄荆坝大桥及连接线项目进展顺利，中心城区建成区面积达85平方公里、常住人口达85万人。完善公建配套设施，五星小学投入使用，市妇幼保健院开工建设。改造棚户区危旧房7120套，中心城区货币化安置率100%，继续走在全省前列。改造老旧院落87个。实施城区交通治堵“五年持久战”，西林大桥南北桥头节点改造实现立体互通，沱江大桥加宽改造按期推进。“城市变景区”成效明显，完成大洲广场提升改造和松山公园建设，花萼湿地、甜蜜花园等景观项目顺利推进。加强城市精细化管理，拆除违法建设11.6万平方米，新(改)建公厕40座。国家卫生城市创建工作步伐加快，“交通安全·文明礼让”行动成效明显。

　　县域经济竞相发展。县(市、区)经济实力明显增强，底部基础不断夯实。市中区作为全国第二批农村改革试验区，顺利通过农业部终期评估，城南新区建设步入快车道，川南大草原、大千欢乐世界等旅游项目建成营业。东兴区现代服务业蓬勃发展，电子商务、现代物流和会展业等新兴业态加快培育，以汉安大道为中轴线的区域商业中心初具雏形。隆昌撤县设市开启发展新篇章，全域旅游初见成效，成功创建国家卫生县城、省级文明城市、省级园林城市，被评为全省“三农”工作先进县。资中县历史文化名城影响力不断扩大，现代农业发展步伐加快，被评为全省县域电子商务十佳县。威远县工业转型发展取得实效，新能源、新材料等新兴产业加快发展，国家级页岩气产业示范园进入规模开采阶段。白马、田家、界市、罗泉、连界等重点镇加快建设，建成幸福美丽新村179个。

　　(三)坚持促改革、扩开放，发展活力持续释放

　　重点领域改革深入推进。系统推进全面创新改革“一号工程”，编制完成“九张清单”，申请专利超过2000件，实施科技重大成果转化项目25个。“600兆瓦超临界循环流化床锅炉技术开发、研制与工程示范”项目荣获国家科学技术进步奖一等奖。成功创建国家高新区，并实现对东兴区部分街道托管。完成国家和省级改革试点任务13项，稳步推进公务员职务与职级并行、生育保险与基本医疗保险合并等全国改革试点。完成141个村集体资产股份制改革，农村改革在全省中期评估考核中位居第二。深化供给侧结构性改革，关闭煤矿9户。“放管服”改革全面深化，压减前置审批事项152项，80%以上公共服务事项实现网上办理。深化商事制度改革，新增市场主体2.2万余户。深化财税金融改革，完成市区财政体制调整，内江农商行正式运行。公共资源交易、供销社、不动产登记、国资国企、科技、统计等领域改革取得实效。

　　开放合作实现突破。借力“中外知名企业四川行”、西部国际投资大会等平台，签订重大招商项目82个。成功举办第八届大千龙舟经贸文化节、第三届川南电商博览会等系列活动，发挥“1+N”投资促进政策体系作用，全市到位市外资金增长12.7%。新引进5亿元以上的产业项目78个，特别是投资55亿元的新能源汽车产业园项目，仅用7个月就完成“对接—签约—开工”，掀开了我市汽车产业发展的崭新篇章。深入实施返乡创业“回家工程”，新增返乡创业人员2916人，创办企业224户，总投资42.8亿元。与成都市签署协同发展战略合作协议，涵盖合作事项34项。与清华大学深圳研究生院、哈尔滨工业大学、西北工业大学等7所院校签订战略合作协议。

　　(四)坚持下深水、动真格，环保力度前所未有

　　流域治理进入国家试点。沱江流域(内江段)获批全国首批、全省唯一的流域水环境综合治理与可持续发展试点。编制完成《内江市沱江流域综合治理和绿色生态系统建设与保护规划(2017—2020年)》，包装重大项目441个，计划总投资1449亿元，其中，水资源综合利用、水污染防治、水生态修复项目65个，计划总投资196亿元。启动实施沱江流域水环境综合治理、城乡垃圾处理设施建设2个PPP项目，总投资超过80亿元。

　　环境突出问题整改有力。顺利通过中央、省环境保护督察，动真碰硬解决环境突出问题，整改完成中央环保督察期间转交办信访件210件、2013—2017年省移交信访投诉件178件、省环保督察发现问题292个、环保部西南督查中心综合督查反馈问题57个，4起环保部挂牌督办的环境违法案件全部完成整改并实现摘牌。实施“10+2”行业性(区域性)环境突出问题专项整治，肥水网箱养鱼等问题得到有效治理，禁养区内规模养殖场关闭搬迁全面完成。立案查处环境违法行为380件，处罚金额2300余万元。

　　区域环境质量得到改善。全覆盖落实河长制，整治完成7条黑臭水体，探索一体式应急治理、微生物修复等治理方法，3个国控断面水质全面改善，濛溪河稳定保持Ⅲ类水质，威远河、球溪河全面消除劣Ⅴ类水质。完成内江城区饮用水取水口上移工程和对口滩饮用水水源地环境保护工程，中心城区集中式饮用水水源地水质全面达标，乡镇集中式饮用水水源地水质达标率提高13.7个百分点。超额完成主要污染物总量减排目标任务，环境空气质量改善幅度居川南第一、全省前列，全年空气优良天数为272天，同比增加11天。农村面源和土壤污染防治积极有效。实施大规模绿化内江行动，人工造林4.9万亩，其中绿化沱江干流1.2万亩，全市森林覆盖率达34.13%。

　　(五)坚持惠民生、促和谐，人民生活更加美好

　　脱贫攻坚再战再捷。突出脱贫攻坚“头等大事”，投入各类扶贫资金13.6亿元，大力实施22个扶贫专项方案，帮助1.9万贫困人口就业，建成易地扶贫搬迁住房1879套，涉及搬迁对象4946人。省级、市级“四好村”分别达100个、502个。“四项基金”和特殊困难救助基金规模达2.3亿元，发放使用1.8亿元，惠及贫困人口8.1万人次。创新建立党建引领扶贫、“定位提醒”和“立体督查”等扶贫机制，解决“插花式”贫困的经验做法获得国务院扶贫办肯定。实现85个贫困村退出、27791名贫困人口脱贫，全市贫困发生率下降到1.28%。

　　民生工程全面完成。民生支出155.4亿元，占一般公共预算支出的71.4%，比全省平均水平高6.4个百分点，“十项民生工程”“20件民生实事”全面完成。开展“甜城双创”活动，全市城镇新增就业5万余人。全面完成城乡基本医疗保险统筹工作，参保人数达到295万人，保险补助标准增加至450元，住院费用政策范围内报销比例达到75%，在全省率先出台100个单病种报销实施方案。城镇低保、农村低保、特困人员和困难残疾人每月生活补贴标准，分别提高至460元、280元、300元和70元。为2.24万名家庭经济困难寄宿制学生发放生活补助，为3.8万名农村义务教育在校学生提供免费营养午餐。新增养老机构床位2935张，新建城乡社区日间照料中心87个，为11.8万名困难家庭失能老人和80周岁以上高龄老人提供居家养老服务。完成农村危房改造2759户。持续开展农村水井水质监测，整改不合格水井1755口，受益人口2万余人。

　　社会事业扎实推进。“十大社会事业惠民工程”进展顺利，市体育中心、市文化中心、内江师院新校区加快建设，内江六中高新校区、市第一人民医院新区、第二社会福利院及精神病康复院完成主体工程，市中医医院西林新区建成投用，创建内江卫生与健康职业学院纳入《四川省高等学校设置“十三五”规划》。新(改)建公办幼儿园17所，学前三年入园率达87.8%。义务教育学校标准化建设全面完成，隆昌市、威远县、市中区建成县域内义务教育基本均衡县。全市高中入学率达88.5%，藏区“9+3”学生初次就业率达100%，高考万人本科上线率保持全省前列，内江职院成功创建省级示范高职院校。全面消除市域内所有地方病。新建113个行政村广播“村村响”、6576户电视“户户通”，建成村文化室85个。承办省级以上大型体育赛事7场次，开展“周末快乐体育”活动近500场次，内江籍运动员在国际国内赛事上取得优异成绩。大力弘扬社会主义核心价值观，“践行十爱·德耀甜城”主题活动扎实有效。完成村(居)委会换届选举。国防教育、国防动员、人民防空、双拥共建、优抚安置等工作取得新进步。外事侨务、对台工作、民族宗教、机构编制、统计调查、气象水文、防灾减灾、检验检疫、无线电管理、保密、档案、史志等工作实现新发展。新闻出版、红十字、慈善、老龄、妇女儿童等事业取得新成绩。

　　社会保持和谐稳定。全市各类生产安全事故起数、死亡人数分别下降42.3%、8.2%，没有发生较大及以上生产安全事故。启动“放心舒心消费城市”创建，实施最严格的食品药品监管，连续13年未发生重特大食品药品安全事故。创新社会治理，深入实施“七五”普法，完善多元化矛盾化解机制，有效化解一批矛盾纠纷和遗留问题，圆满完成党的十九大等重大节点维稳信访安保工作，实现了“六个不发生”。深化公安、司法行政体制改革，完成76个司法所规范化建设，开工建设6个城区派出所。及时妥善处置各类突发事件，应急处置能力明显提升。推进“平安内江”建设，依法打击各类违法犯罪活动，深入开展禁毒、打击“盗抢骗”等专项行动，公众安全感满意率保持在95%以上。

　　(六)坚持转作风、提效能，自身建设全面加强

　　深入学习贯彻党的十九大精神，牢固树立“四个意识”，坚定以习近平新时代中国特色社会主义思想武装头脑、指导实践、推动工作。推进“两学一做”学习教育常态化制度化，认真落实中央八项规定精神和省、市十项规定要求，“三公经费”同比下降22.8%。加强与人大代表、政协委员的联系，自觉接受人大政协监督，办理人大代表议案1件，建议、批评和意见185件，办理政协委员提案385件。广泛听取民主党派、工商联、无党派人士、人民团体的意见。积极支持工会、共青团、妇联、科协、残联、侨联等群团组织开展工作。大力推进依法行政，参与制定我市第一部地方性法规《内江市甜城湖保护条例》，坚持政府常务会会前学法，完善法律顾问制度，全面实施重大行政决策合法性审查和规范性文件备案审查。规范行政处罚裁量权，强化行政复议、应诉、调解和仲裁工作。推进政务公开，实施“三张清单”制度，全面推行“双随机、一公开”监管，全市行权平台办件128万件，行政权力公开运行率超过95%。完成市级政府投资工程决算审计项目28个，审减资金2.24亿元。坚持“一岗双责”，严格落实党风廉政建设责任制，大力整治侵害群众利益的不正之风和腐败问题，政纪处分195人。

　　各位代表，过去一年，遇到的困难比预料的多，面临的挑战比预料的大。我们坚持抓早、抓紧、抓主动，出台稳定经济增长系列措施，经济总量不断增加;我们加快转方式、调结构、促升级，大力发展高新技术和战略性新兴产业，经济结构出现积极变化;我们昼夜兼程、紧盯不放，引进一批重大项目，实现产业招商重大突破;我们积极应对财政金融新形势，努力破解资金、土地等要素瓶颈，推动“项目年”部署落地落实;我们认真打好污染防治“三大战役”，持续整治环境突出问题，生态环境明显改善;我们聚焦群众关心的重要问题，圆满完成脱贫攻坚年度目标任务，办成一批民生实事，人民群众获得感有所增强。这些成绩的取得，是市委统揽全局、科学决策的结果，是市人大、市政协监督支持、共谋发展的结果，是全市干部群众同心图强、埋头苦干的结果!在此，我代表市人民政府，向420万内江人民，向人大代表、政协委员、各民主党派、工商联、无党派人士、各人民团体，向驻内部队、武警官兵、政法干警和民兵预备役人员，向关心、支持、参与内江建设发展的各界朋友，表示诚挚的感谢!

　　在总结成绩的同时，我们也更加清醒地认识到，内江经济社会发展还存在不少困难和挑战，地区生产总值、社会消费品零售总额、全社会固定资产投资等未能实现预期目标。发展不平衡不充分，经济总量不大，产业结构不优，创新能力不强，部分行业企业发展困难。财政收入占经济总量比重偏低，税源培植亟待加强。农村基础设施薄弱，乡村振兴任重道远。城市综合配套不够完善，交通拥堵还未根本解决，创建国家卫生城市还需攻坚破难。精准脱贫任务艰巨，饮水安全、教育、医疗等与群众美好生活需要还有差距。少数政府部门和公务人员担当意识不强、办事效率不高。对此，我们将以对人民负责、对岗位负责、对脚下这片土地负责的态度，采取有力措施，认真加以改进。

二、2018年工作安排

　　2018年是贯彻党的十九大精神的开局之年，是改革开放40周年，是决胜全面建成小康社会、实施“十三五”规划承上启下的关键一年。今年政府工作的总体要求是：以习近平新时代中国特色社会主义思想为指导，深入贯彻党的十九大和中央经济工作会议精神，认真落实省第十一次党代会、省委十一届二次全会、省委经济工作暨全省金融工作会议精神，按照市委七届六次全会、市委经济工作暨全市金融工作会议部署，坚持稳中求进工作总基调，坚持新发展理念，紧扣社会主要矛盾变化，按照高质量发展要求，统筹推进“五位一体”总体布局和协调推进“四个全面”战略布局，认真落实市委“一个总体取向、两个率先、三个基本建成、三大发展战略、四个主动融入、五个重大支撑”的总体发展思路，坚定推进供给侧结构性改革，统筹推进稳增长、促改革、调结构、惠民生、防风险各项工作，推动质量变革、效率变革、动力变革，打好防范化解重大风险、精准脱贫、污染防治三大攻坚战，促进经济社会持续健康发展。

　　今年全市经济社会发展的主要预期目标是：地区生产总值增长7.6%左右;全社会固定资产投资同口径增长10.0%;社会消费品零售总额增长11.0%;地方一般公共预算收入同口径增长7.8%;城乡居民人均可支配收入分别增长8.5%、9.0%;居民消费价格指数涨幅控制在3.0%以内;完成省下达的节能减排和环境保护目标任务。

　　围绕总体要求和预期目标，我们将重点抓好以下工作。

　　(一)打好“三大攻坚战”，夯实高质量发展基础

　　防范化解重大风险。加强金融监管，严厉打击非法集资活动，稳妥有序推进互联网金融风险专项整治。防范化解企业债务风险，引导有条件的企业进行市场化法治化债转股，降低企业杠杆率。防范化解房地产市场风险，强化诚信体系建设，推动行业健康平稳发展。防范化解政府债务风险，严格债务限额管理，有效化解存量债务。深入开展社会稳定风险评估，妥善化解各类社会矛盾，依法严厉打击各类违法犯罪活动，坚决打赢扫黑除恶专项斗争，维护社会稳定。加强重点行业和人员密集场所的安全监管，持续开展“打非治违”行动，加大隐患排查整治力度，严防重特大事故发生。加强食品药品监管，创建国家食品安全示范城市，守住群众“舌尖上”的安全。

　　切实抓好精准脱贫。坚定不移把脱贫攻坚作为“头等大事”，确保88个贫困村退出、25349名贫困人口脱贫，完成易地扶贫搬迁3068人。深入开展“五个一批”脱贫攻坚行动，大力实施22个扶贫专项方案。实施“雨露计划”“栋梁工程”，确保全市义务教育阶段的贫困学生无一人因贫辍学。落实“十免四补助”“八个百分之百”等政策，继续向贫困群众提供免费健康体检，确保贫困人口在县域内住院费用自付比例控制在10%以内。大力实施“农村家庭能人”培养计划，确保每户有劳动能力的贫困家庭至少有1人就业。扎实开展精神扶贫十大行动，引导贫困群众自力更生、艰苦奋斗。狠抓移民后期扶持，全面完成贫困移民解困任务。强化“回头看”“回头帮”，巩固脱贫成效。

　　强力推进污染防治。持续实施污染防治“三大战役”81个重点项目，加快建设内江市第二污水处理厂、威远县第二污水处理厂、资中经开区污水处理厂、隆昌经开区污水处理厂等污水处理设施，加快推进内江市生活垃圾处理中心项目，确保内江医疗废物处理中心建成投用。打好蓝天保卫战，编制实施《内江市大气环境质量限期达标规划》，大力实施“减排、压煤、抑尘、控秸、治车”五大工程，中心城区PM2.5年均浓度控制在52.9微克/立方米以内，空气质量优良率超过74.4%。打好碧水保卫战，全面落实河长制，完成中心城区4条黑臭水体整治，加大饮用水水源保护区整治力度，县级以上城市和乡镇现用集中式饮用水水源地水质达标率分别达100%和75%。打好净土保卫战，保持土壤环境质量总体稳定。全力抓好环保督察反馈问题整改，系统整治“10+2”行业性(区域性)环境问题。对2个县(市、区)开展市级环保督察。做好第二次全国污染源普查工作。

　　(二)实施乡村振兴战略，开创“三农”工作新局面

　　积极发展现代农业。按照产业兴旺、生态宜居、乡风文明、治理有效、生活富裕的总要求，科学制定乡村振兴战略规划。深入推进“12345”现代农业产业提升行动、“351”特色农业产业发展方案和农业供给侧结构性改革12大工程，扩大“甜城味·大千故里优质农产品”市域公用品牌影响力，深入实施100万头“内江黑猪”产业化项目，加快建设30万亩稻田综合种养产业带，大力培育百里休闲农业产业带，持续推进优质粮油等4大功能区建设，巩固提升5个万亩以上产业示范片，建设农业重点镇30个、特色村215个、专业村200个、精品村100个，加快建设成渝经济区绿色优质农产品基地。严守耕地保护红线，确保全市耕地保有量不低于26.28万公顷、永久基本农田保护面积不低于21.11万公顷。加强永久基本农田划定成果运用，强化高标准农田建设。落实粮食安全行政首长责任制，确保区域粮食安全。

　　统筹建设现代农村。推进7个田园综合体建设，建成幸福美丽新村200个，创建省级“四好村”90个、市级“四好村”255个。统筹推进“四好农村路”建设，加快农村客货运输网络化进程，新(改)建农村公路300公里，开工建设渡改桥3座。加快“五小水利”工程建设，除险加固病险水库8座，新增高效节水灌溉面积1.2万亩。加大中低压配电网改造力度，完成农村电网改扩建项目163个。大力整治农村面源污染，全市行政村生活垃圾有效治理率保持在90%以上。健全乡村治理体系，开展村民自治试点。加强农村人才队伍建设，培养造就一支懂农业、爱农村、爱农民的“三农”工作队伍。

　　全力促进农民增收。加快培育新型农业经营主体，示范发展种养大户200个、家庭农场50户、农民合作社20个、龙头企业5户。开发农业多种功能，发展休闲农业、乡村旅游等新产业新业态，持续增加农民收入。巩固和完善农村基本经营制度，研究落实第二轮土地承包到期后再延长30年的具体措施，推动承包地“三权分置”，确保适度规模经营率提高1.5个百分点。

　　(三)筑牢产业支撑，着力建设经济强市

　　推进工业转型升级。更加重视实体经济发展，深入实施先进制造强市“三百行动”，新发展先进制造企业30户、技改传统制造企业30户、培育制造业名优品牌30个。运用新技术、新工艺、新设备，改造提升特色优势产业，确保完成技改投资160亿元以上。加快实施工业转型升级创新发展三年行动计划，重点发展“四新一大”产业。培育工业经济增量，加快建设新能源汽车产业园、教育信息安全产业园，竣工投产恒通动物制剂生产基地等项目。推进企业梯次培育，新增规模以上工业企业30户，支持更多企业上市。继续落实“工业强市八条措施”，帮助停产半停产企业恢复生产。大力培养引进创新型和高技能人才，打造职业化企业家队伍，让内江成为创业者的乐园、企业家的摇篮。

　　推动服务业扩量提质。深入实施服务业品牌培育“552”工程，新增限额以上商贸企业和规模以上服务业企业20户。实施电子商务集群网状发展“十大工程”，加快建设内江电商产业园、川南电商中心等电商集聚区，壮大行业垂直电商交易平台，继续走在全省前列。实施川南快递物流园、川南邮政物流集散中心等项目，探索推进“川菜中央厨房”建设，引进一批知名物流企业，加快建设现代物流创新发展城市。深入实施旅游发展“一三八”计划，打造“山水之旅、文化之旅、美食之旅”三大品牌，推进石板河、罗泉古镇等项目建设，力争创建国家AAAA级景区1个，旅游总收入增长16%以上。大力发展金融业，新增金融机构或准金融机构3家，实现金融业增加值52亿元;提升金融支持实体经济水平，新增银行业金融机构本外币贷款余额70亿元，新增社会融资120亿元。大力发展会展经济，实施餐饮业提升工程，积极组织促销活动，扩大健康养老、教育培训、文化体育等消费供给，拓展共享经济、体验经济等消费热点。

　　提升园区承载能力。做大做强内江经开区，优化提升以汽车制造、电子信息、生物医药为主，以现代服务业为辅的特色产业发展，努力打造西部地区极具活力和竞争力的国家级经开区。推动内江高新区在新体制下高效运转，完善园区规划，加快产业集聚、基础提升，打造内江经济新的增长极。加强国家农业科技园区建设，确保顺利通过国家复核验收。同步加大市中区经开区、资中经开区、连界工业园等重点园区建设力度，确保全市工业园区新增承载面积2平方公里以上，产业集聚度提高1个百分点。

　　(四)筑牢基础支撑，不断增强发展后劲

　　持续扩大投资规模。落实“项目年”部署，坚持定期举行项目集中开工活动，抓好20余个省级重点项目、300个左右的市级重点项目建设。加快建设“一带一轴一区”重要交通枢纽，完成交通基础设施建设投资20亿元左右。推动川南城际铁路、连乐铁路、蓉昆高铁(内江段)等项目建设。开工建设椑木东互通连接线、高桥互通连接线，加快建设内江城市过境高速公路;力争开工建设资威路快速通道，继续开展东泸路、资荣路、内威路、资东路和内江至南溪(含内江至大足段)高速公路前期工作，推动井研至资中至乐至高速公路纳入省高速公路路网规划。完善水利基础设施，建成东兴区联合水库，加快建设长桥水库、两河口水库，大力实施大中型灌区续建配套与节水改造，开工建设向家坝灌区北总干渠一期内江管线工程，完成水利基础设施建设投资10亿元以上。加快推进无线通信网络建设，扩大4G网络覆盖面。强化要素保障，发挥地方债券和产业发展基金作用，用好PPP投融资模式，切实解决好征地拆迁中的具体问题，保障重大项目顺利实施。

　　加快建设“双百”城市。强化规划引领，做好重要节点的城市设计。加大投入力度，实施“双百”城市项目200个左右。拓展城市空间，加快新城“一中心四组团”建设，打通组团间连接通道，建成汉渝大道跨内宜高速跨线桥、北环线五段、林荫大道，确保中心城区建成区面积达90平方公里、常住人口达90万人。完善城市功能，打通12条城市断头路，开工建设10个停车场、2个加气站、3个示范充电站桩等配套设施，建成内江综合客运中心站，完成沱江大桥加宽改造。优化人居环境，启动桂湖街、胜利街道城中村等棚户区改造，全市改造棚户区危旧房7749套;推动“城市变景区”，完成人民公园改造提升和千水湖公园、玉峰山公园、谢家河公园北段建设。坚持建管并重，强力拆除违法建设，加快建设智慧城市，提高精细化管理水平。

　　壮大县域经济实力。注重分类指导县域经济发展，努力建设特色经济强县。支持市中区拓展城市空间，继续开发城南新区，打造成渝乡村旅游样板区。支持东兴区有序建设椑木新城、东兴南部新城，做大做强西南循环经济产业园区、内江长江现代农业园区、东兴现代服务业产业园区。支持隆昌市建设全省工业强县示范县、全省现代农业示范县，依托省级宜居县城试点，建设城湖一体、景城相融的宜居县城。支持资中县农业产业提升、工业延链转型、电商扩面增效，有序推进县城拓展，增添历史文化名城新活力。支持威远县加快建设县城新区，开发穹窿地貌等特色旅游产业，加快产业转型升级，推动工业大县向经济强县转变。支持各县(市、区)持续抓好17个国省重点镇、6个全省“十三五”特色小镇建设，打造一批加工制造、现代农业、休闲旅游、科教文化等特色小镇。

　　(五)筑牢动力支撑，厚植经济发展新优势

　　推进全面创新改革。加快建设区域性科技创新中心，提升科技企业孵化器功能，入驻企业和研发机构突破430家。完善六大科技公共服务平台，推动川南检验检测共享平台和技术交易平台深度融合发展。新签订市校、校企合作协议50个以上，推动已签约项目落地落实，实施重大科技成果转化项目20项以上。新培育科技型中小企业100户。举办 “高新杯”创客大赛等活动，打造内江“双创”活动品牌。

　　深化供给侧结构性改革。持续推进“三去一降一补”，稳妥处置“僵尸企业”，大力发展住房租赁市场，加快建立多主体供给、多渠道保障、租购并举的住房制度，扩大农民工纳入住房公积金覆盖范围。落实富余电、直购电政策，推动天然气转供变直供，加大减税降费力度，降低实体经济生产经营成本。推进质量强市，抓好“一提一创一培”，打造一批具有区域竞争力的品牌、企业和产品。

　　统筹推进重点领域改革。深化农村集体产权制度改革，完成全国第二批农村改革试验区拓展试点、全省农村综合改革试验区、全省新型职业农民制度试点等改革任务。完成公务员职务与职级并行、生育保险与基本医疗保险合并等全国改革试点任务。强化“放管服”改革，优化权力事项目录，动态调整责任清单;深化“互联网+政务服务”，推进大数据中心建设，整合共享各部门数据资源;协同推进商事制度改革，营造公平营商环境，新发展市场主体1.4万户。深化财税金融体制改革，加快资中农商行筹建工作。深化统计管理体制改革，搞好第四次全国经济普查。同步推进教育、卫生、科技、公共资源交易、国资国企、事业单位、供销社、生态体制等重点领域改革。

　　提升开放合作水平。坚持精准招商，破解招商瓶颈，确保到位市外资金增长10%以上。积极参加“第十七届西博会”“第六届四川农博会”“中外知名企业四川行”“千企行丝路”等重大活动，办好川南电商博览会、大千龙舟经贸文化节等活动，加快引进一批优质项目。继续实施返乡创业“回家工程”，新增返乡创业人员1000人、企业100户，完成投资30亿元以上。做好签约项目跟踪服务，确保省政府督办项目履约率、开工率、资金到位率分别超过90%、80%、30%。强化“四个主动融入”，提升与成都、重庆的产业协作水平，打造成渝经济区投资洼地。突出与川南城市的合作，促进产业互补、交通互联、民生互惠。

　　(六)筑牢生态支撑，建设美丽四川先行区

　　深化沱江流域综合治理。以内江沱江流域综合治理和绿色生态系统建设与保护“世代工程”为统揽，稳步推进重点流域水环境综合治理与可持续发展国家试点。开工建设沱江流域水环境综合治理、城乡垃圾处理设施建设2个PPP项目，加快建设智慧环保。强化“一增一减一治”，加强自然保护区管理和天然林保护。加快创建国家园林城市和省级森林城市，扎实推进“八大绿化行动”，完成造林4.23万亩，力争全市森林覆盖率达到34.35%。

　　倡导绿色生产生活方式。培育壮大节能环保、清洁生产、清洁能源产业，推动绿色低碳循环发展。推进燃煤小锅炉淘汰和改造升级，开展重点企业节能节水行动。实施农药化肥零增长行动，秸秆综合利用率达85%，启动资中县全国畜禽粪污综合利用整县推进试点。开展创建绿色家庭、绿色学校、绿色社区等行动，政府机关和公共服务单位带头使用新能源汽车。增强全民环保意识，形成绿色发展人人有责、人人参与、人人共享的风尚。

　　健全生态环境保护机制。全面落实环境保护“党政同责、一岗双责”主体责任，认真实施《内江市甜城湖保护条例》，严格落实主体功能区规划，坚决守住生态保护红线。严格环境准入，优化规划环评和项目环评。强化源头防控，健全企业排污许可、环境信用评价、信息强制性披露等制度。建立生态环境损害赔偿制度，强化监管执法，坚决制止和惩处破坏生态环境行为。

　　(七)筑牢民生支撑，提升人民群众获得感

　　深入实施民生工程。大力推进“十项民生工程”“20件民生实事”，统筹实施“内江民生十有行动”，做到民生支出占一般公共预算支出比重不低于全省平均水平。把就业作为民生之本，新增城镇就业3.7万人，城镇登记失业率控制在4.1%以内。为2万名家庭经济困难寄宿制学生发放生活补助，为3.8万名农村义务教育在校学生提供免费营养午餐。实行城乡医疗救助一站式服务，加大重特大疾病救助力度。适时提高低保、特困人员供养和困难残疾人生活补贴标准，建立照料特困人员护理补贴制度。为65岁以上老人提供免费健康体检，继续为困难家庭失能老人和80周岁以上高龄老人提供居家养老服务。鼓励社区和社会力量开办养老机构，完成农村公办适老化改造床位1300张。推进“厕所革命”，新(改)建公共厕所200座;新(改)建标准化农贸市场21个、便民服务点8个，确保创建国家卫生城市通过省级评估推荐。创建“放心舒心消费城市”，培育打造示范点100个。巩固提升农村饮水安全，新增受益人口3.2万人。改造农村危房5800户。

　　用心办好社会事业。加快推进“十大社会事业惠民工程”，加快内江师院新校区、市体育中心、市文化中心、内江大剧院建设，基本建成内江卫生与健康职业学院，确保市第一人民医院新区、内江六中高新校区投入使用。坚持教育优先，新(改)建公办幼儿园13所，实现全市县域内义务教育基本均衡，抓好中考改革和高中新课程、新高考改革，加快发展职业教育，推进中高职衔接，推动高等教育改革发展。推进分级诊疗，支持社会办医，发展中医药大健康产业。传承和弘扬中华优秀传统文化，扎实开展“践行十爱·德耀甜城”主题活动，持续开展群众文化活动，推进“书香内江”建设。积极举办体育赛事，组织开展群众体育活动，大力推进“三大球”进校园，积极参加省第13届运动会。同时，扎实做好国防动员、统计调查、民族宗教、外事侨务、对台工作、友协、保密、档案、史志等工作，大力发展妇女儿童、老龄、红十字和慈善等事业。

　　切实加强社会治理。完善社会治安防控体系，加强信访法治化建设。深化公安、司法行政体制改革，完成市县综治中心、市工读学校、派出所、司法所规范化建设。做好流动人口和特殊人群服务管理。推进依法治市，扎实开展“七五”普法活动，持续推进“法律七进”。健全协调联动和快速反应机制，提高应急处置能力。做好地灾防治、防汛抗旱和森林防火工作。

三、新时代展现政府新作为

　　新时代要有新气象，更要有新作为。站在新的历史起点，我们深知，人民对政府工作寄予厚望。我们将不断提升政府服务水平，积极回应群众期盼，努力建设人民满意政府。

　　(一)不忘初心，牢记使命。严守政治纪律和政治规矩，牢固树立“四个意识”，坚决维护以习近平同志为核心的党中央权威和集中统一领导，坚决维护习近平总书记的核心地位。坚定理想信念，认真开展“不忘初心、牢记使命”主题教育，大力弘扬“红船精神”。坚持以人民为中心的发展思想，牢记为人民服务的根本宗旨，始终做到听民声、察民情、解民忧。大兴调查研究之风，大力解决群众最关心、最直接、最现实的利益问题。清理取消不合法不合理的证明和手续，着力解决群众办证难、办事难问题。

　　(二)埋头苦干，创造一流。夙夜在公、昼夜兼程，践行“立说立行、马上就办”，静下心来干事业、扑下身子抓落实。崇尚实干、拒绝空谈，发扬“钉钉子”精神，对认准的事情，一抓到底、不胜不休。勇于担当、敢于负责，做到在问题面前不回避、困难面前不躲闪、挑战面前不畏惧。转变职能、提高效率，坚决整治不作为、慢作为、假作为、乱作为，努力打造一支行动迅速、善抓落实、勤政为民的公务员队伍。不甘人后、奋勇争先，破除思想观念束缚、体制机制障碍、方式方法制约，敢当改革先行者，争当干事急先锋，争取更多工作走在全国全省前列。坚持“三个区分开来”，健全激励机制和容错纠错机制，旗帜鲜明为敢于担当、踏实做事、不谋私利的干部撑腰鼓劲。

　　(三)依法行政，廉洁自律。自觉接受人大政协监督，认真办理人大代表建议和政协委员提案，主动听取民主党派和社会各界的意见，支持监察监督和司法监督。坚持常务会会前学法，完善法律顾问制度，严格按法定程序决策，加快制定地方政府规章，配合制定《内江市城市园林绿化条例》等地方性法规。深化政务公开，加强行政复议和行政应诉，切实提高政府公信力。强化廉政风险防控，认真落实党风廉政建设责任制，深入贯彻中央八项规定实施细则精神和省、市十项规定实施细则，坚决防止“四风”问题反弹回潮。扎实开展政策落实跟踪审计、领导干部自然资源资产离任审计和经济责任、财政、投资等专项审计，推进审计监督全覆盖。以顽强的意志品质，以零容忍的态度，严肃查处侵害群众利益的不正之风和腐败问题，展现清廉政府新形象。

　　各位代表，让我们更加紧密地团结在以习近平同志为核心的党中央周围，在省委、省政府和市委的坚强领导下，紧紧依靠全市人民，讲法治、重实干、敢担当、守规矩，为加快建设幸福美丽内江而努力奋斗!
